# Supplementary material for: Decoding Codon Bias: The Role of tRNA Modifications in Tissue-Specific Translation
Source: Int J Mol Sci. 2025 Jan 15;26(2):706. doi: 10.3390/ijms26020706 (PMC11766445; doi:10.3390/ijms26020706)

Supplementary tables legends:

Supplementary table S1: Normalized peak areas of RNA modifications detected by LC-MS/MS. Related to figure 1.

Supplementary table S2: Differentially translated genes from the Ribo-seq dataset. Related to figure 3.

Supplementary table S3: Buffer gradient used for LC-MS/MS analysis.

Supplementary table S4: MRM table used for LC-MS/MS analysis.

Supplementary table S5: Sequences for EGFP and mutated EGFP used for AAV vector design

Supplementary figure S1: a: A compiled chromatogram showing all the detected modifications and their relative intensities. b: Variable (modifications) plotting from PLS-DA analysis. c: VIP (variable importance) plot from the PLS-DA analysis.

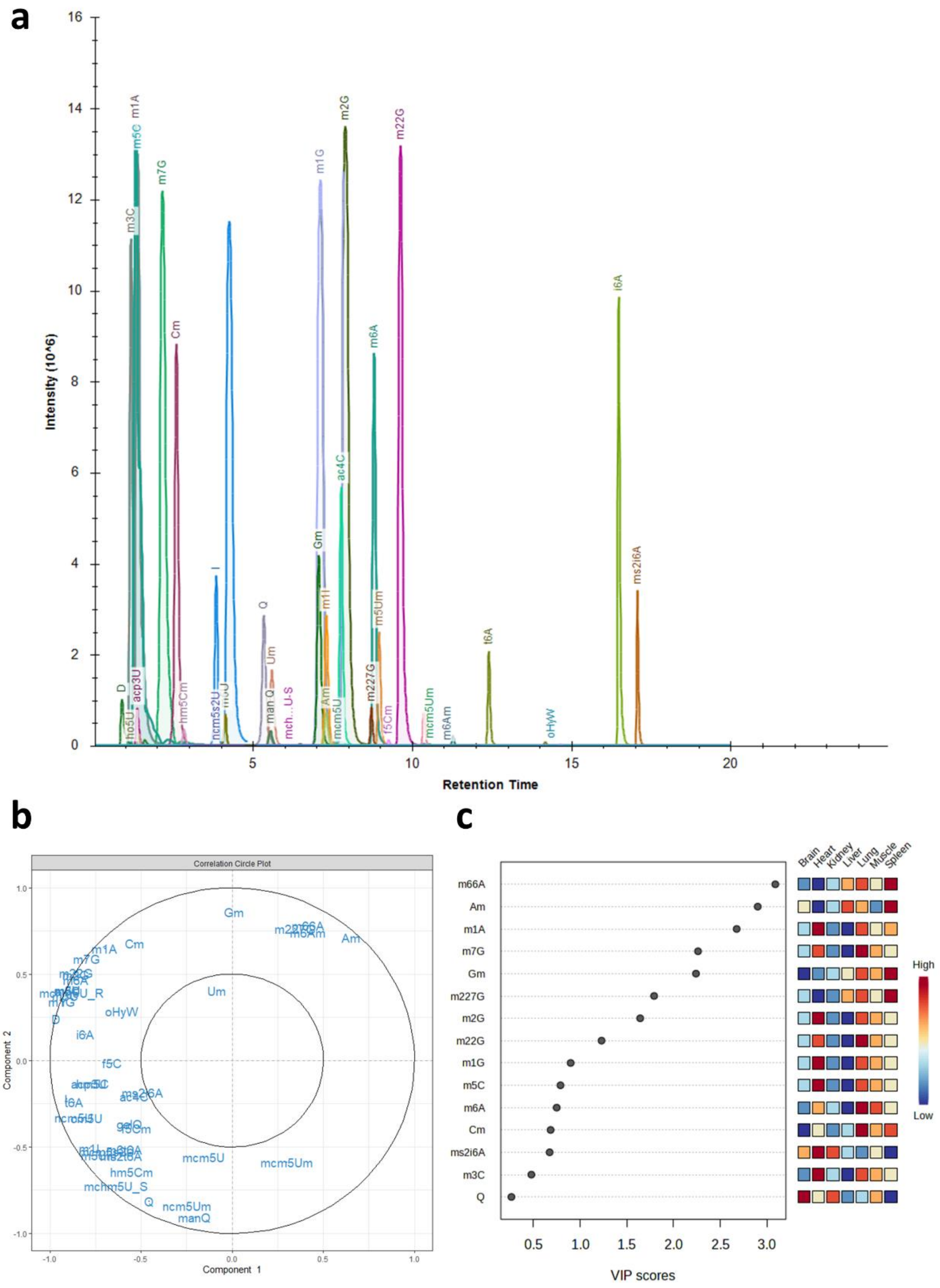

Supplementary figure S2: Detailed analysis of modifications exclusively expressed in tRNA. Y-axes represent normalized peak areas. One-way ANOVA was conducted with Turkey's post-hoc analysis. *: $p < 0.05$, **: $p < 0.005$, ***: $p < 0.0005$, ****: $p < 0.0001$.

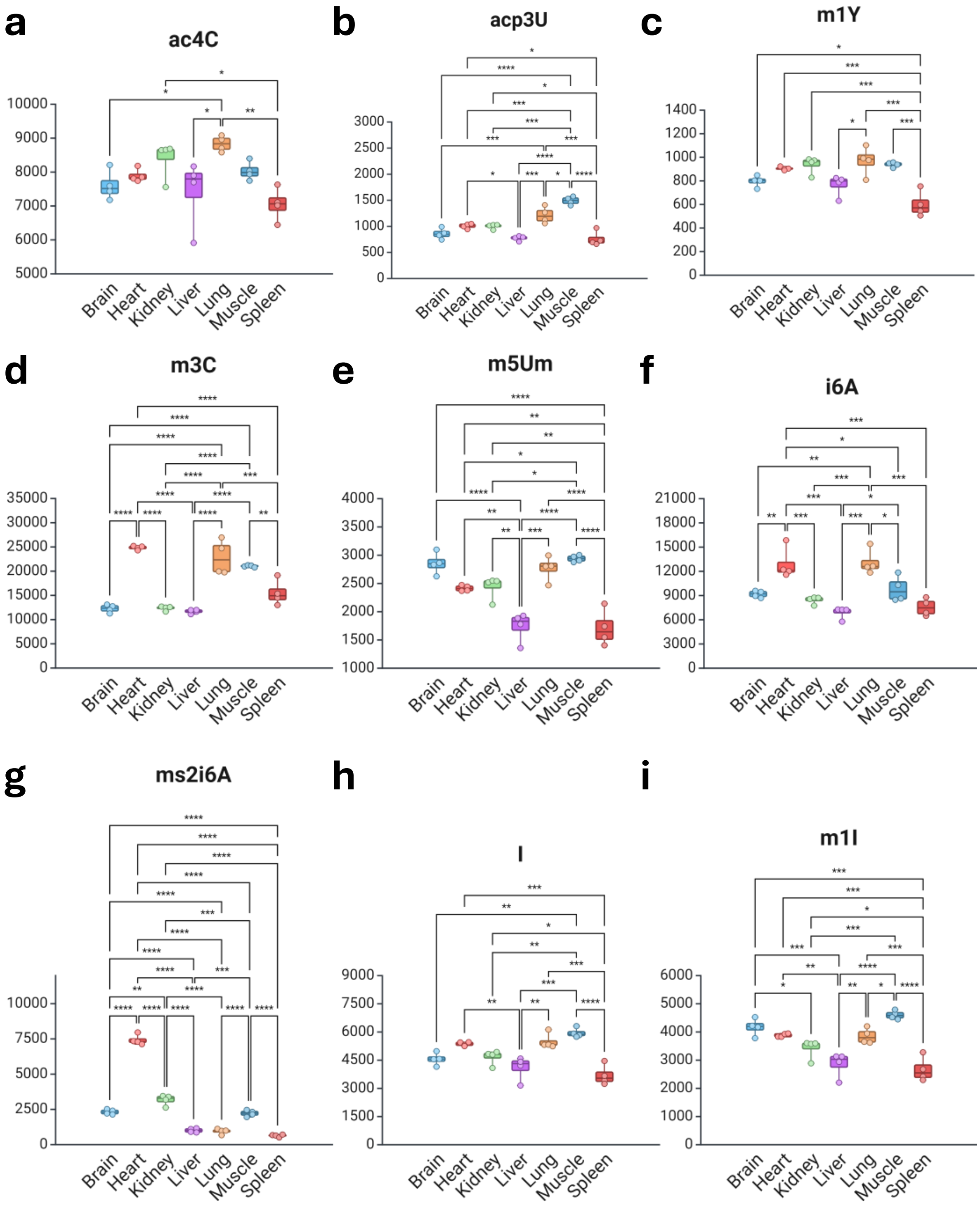

a
ac4C
10000
9000
8000
7000
6000
5000
b
acp3U
3000
2500
2000
1500
1000
500
0
c
m1Y
1400
1200
1000
800
600
400
200
0
d
m3C
35000
30000
25000
20000
15000
10000
5000
0
e
m5Um
4000
3500
3000
2500
2000
1500
1000
f
i6A
21000
18000
15000
12000
9000
6000
3000
0
g
ms2i6A
10000
7500
5000
2500
0
h
I
9000
7500
6000
4500
3000
1500
0
i
m1I
6000
5000
4000
3000
2000
1000
0
Brain
Heart
Kidney
Liver
Lung
Muscle
Spleen

Supplementary figure S3: Detailed analysis of two sets of tRNA anticodons modifications. Q and its derivatives manQ and galQ as well as t6A and its derivatives ms2t6A and m6t6A. Y-axes represent normalized peak areas. One-way ANOVA was conducted with Turkey's post-hoc analysis. *: $p < 0.05$, **: $p < 0.005$, ***: $p < 0.0005$, ****: $p < 0.0001$.

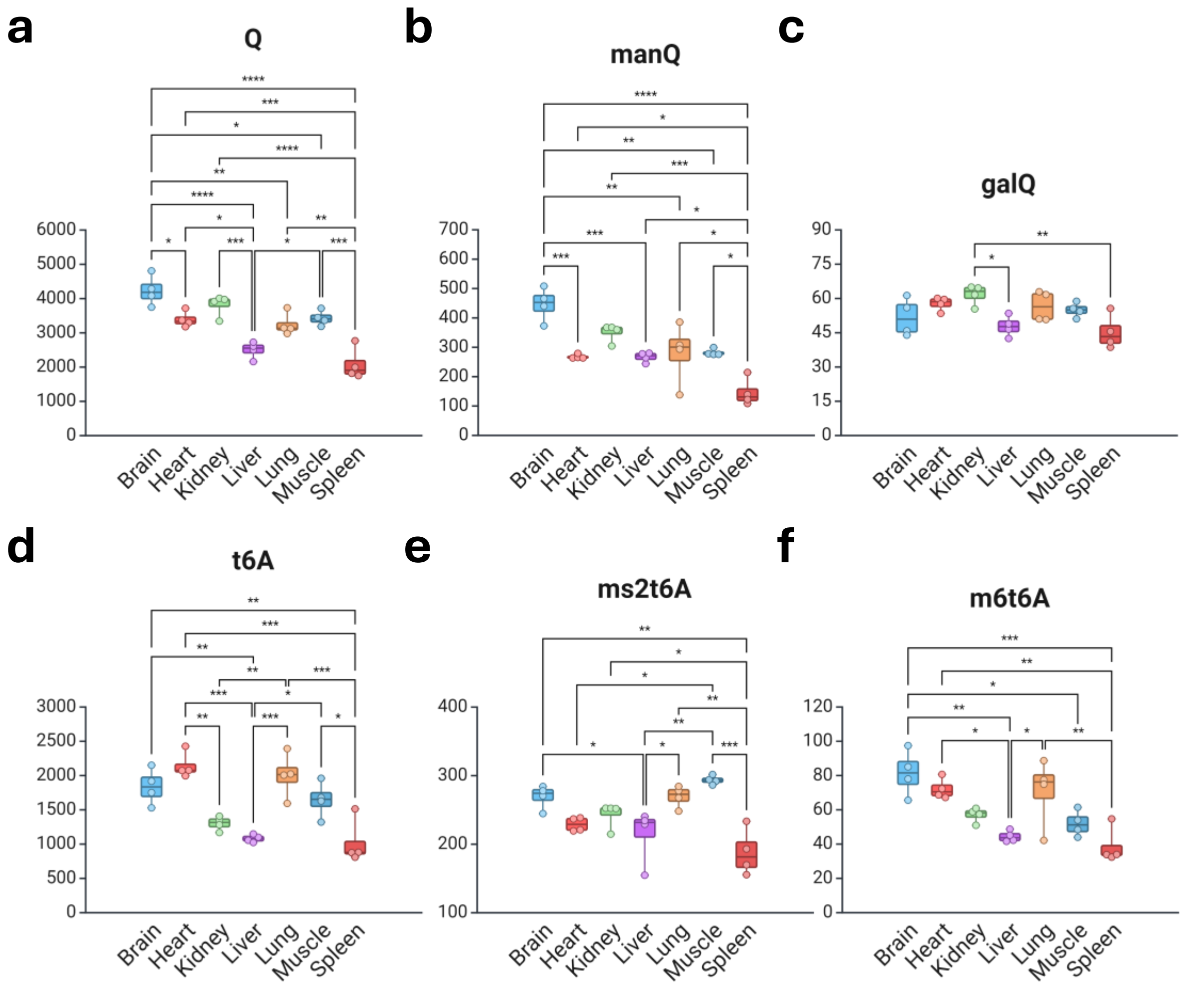

a
Q
6000
5000
4000
3000
2000
1000
0
b
manQ
700
600
500
400
300
200
100
0
c
galQ
90
75
60
45
30
15
0
d
t6A
3000
2500
2000
1500
1000
500
0
e
ms2t6A
400
300
200
100
f
m6t6A
120
100
80
60
40
20
0
Brain
Heart
Kidney
Liver
Lung
Muscle
Spleen

Supplementary figure S4: Detailed analysis of modifications related to the activity of ALKBH1 enzyme, a tRNA dioxygenase enzyme. **a & b:** Synthesis pathway for hm5C, hm5Cm, f5C, and f5Cm modifications. In the cytosol (**a**), NSUN2 methylates cytosine at position 34, which is further converted to hm5C and f5C by ALKBH1. hm5C and f5C are further 2'O-ribose methylated by FTSJ1. In mitochondria (**b**), FTSJ1 is not present. Y-axes represent normalized peak areas. One-way ANOVA was conducted with Turkey's post-hoc analysis. *: $p < 0.05$, **: $p < 0.005$, ***: $p < 0.0005$, ****: $p < 0.0001$.

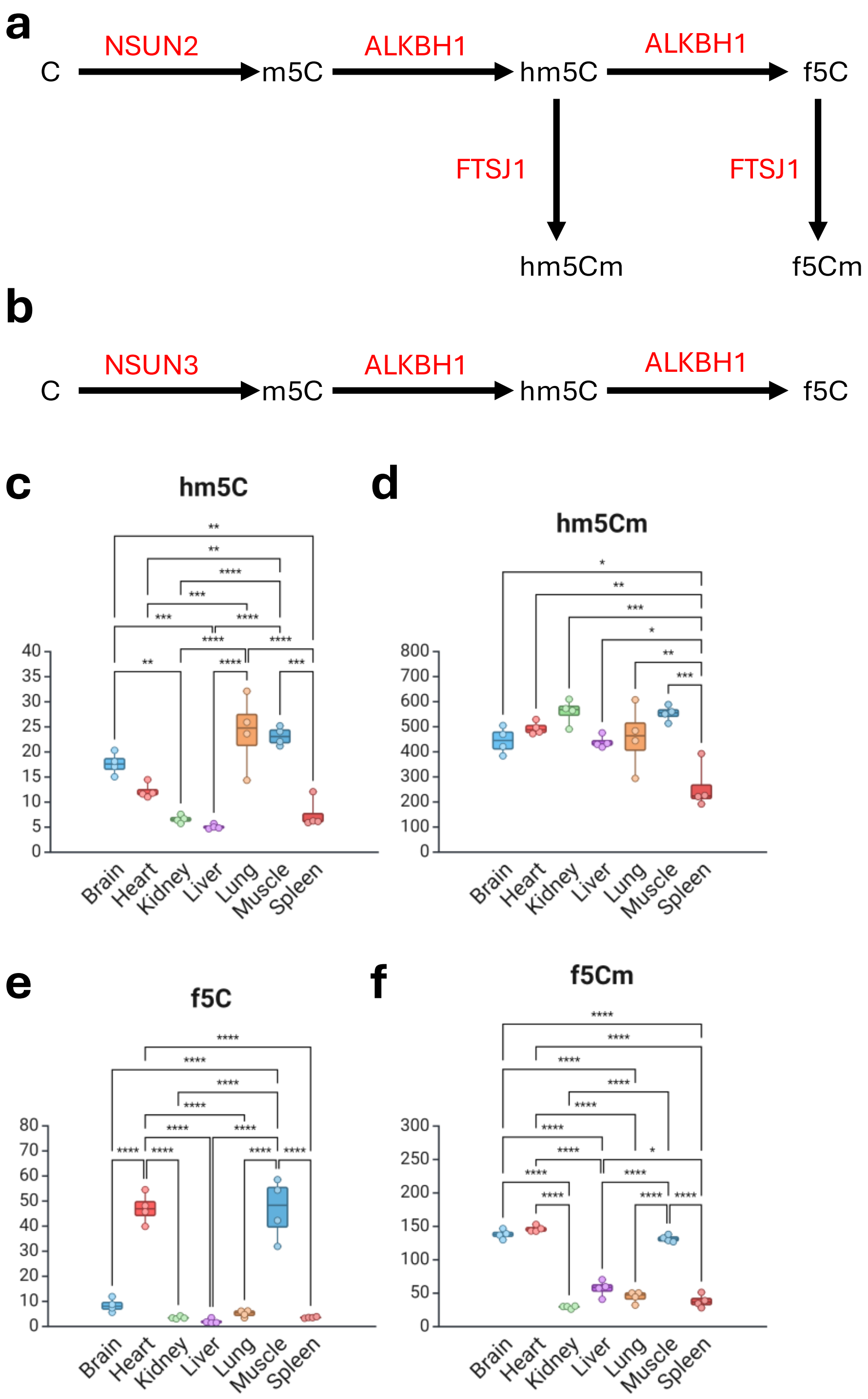
a
C
NSUN2
m5C
ALKBH1
hm5C
ALKBH1
f5C
FTSJ1
hm5Cm
FTSJ1
f5Cm
b
C
NSUN3
m5C
ALKBH1
hm5C
ALKBH1
f5C
c
hm5C
d
hm5Cm
e
f5C
f
f5Cm
Brain
Heart
Kidney
Liver
Lung
Muscle
Spleen

Supplementary figure S5: Detailed analysis of tRNA anticodons modifications. Y-axes represent normalized peak areas. One-way ANOVA was conducted with Turkey’s post-hoc analysis. *: $p < 0.05$, **: $p < 0.005$, ***: $p < 0.0005$, ****: $p < 0.0001$.

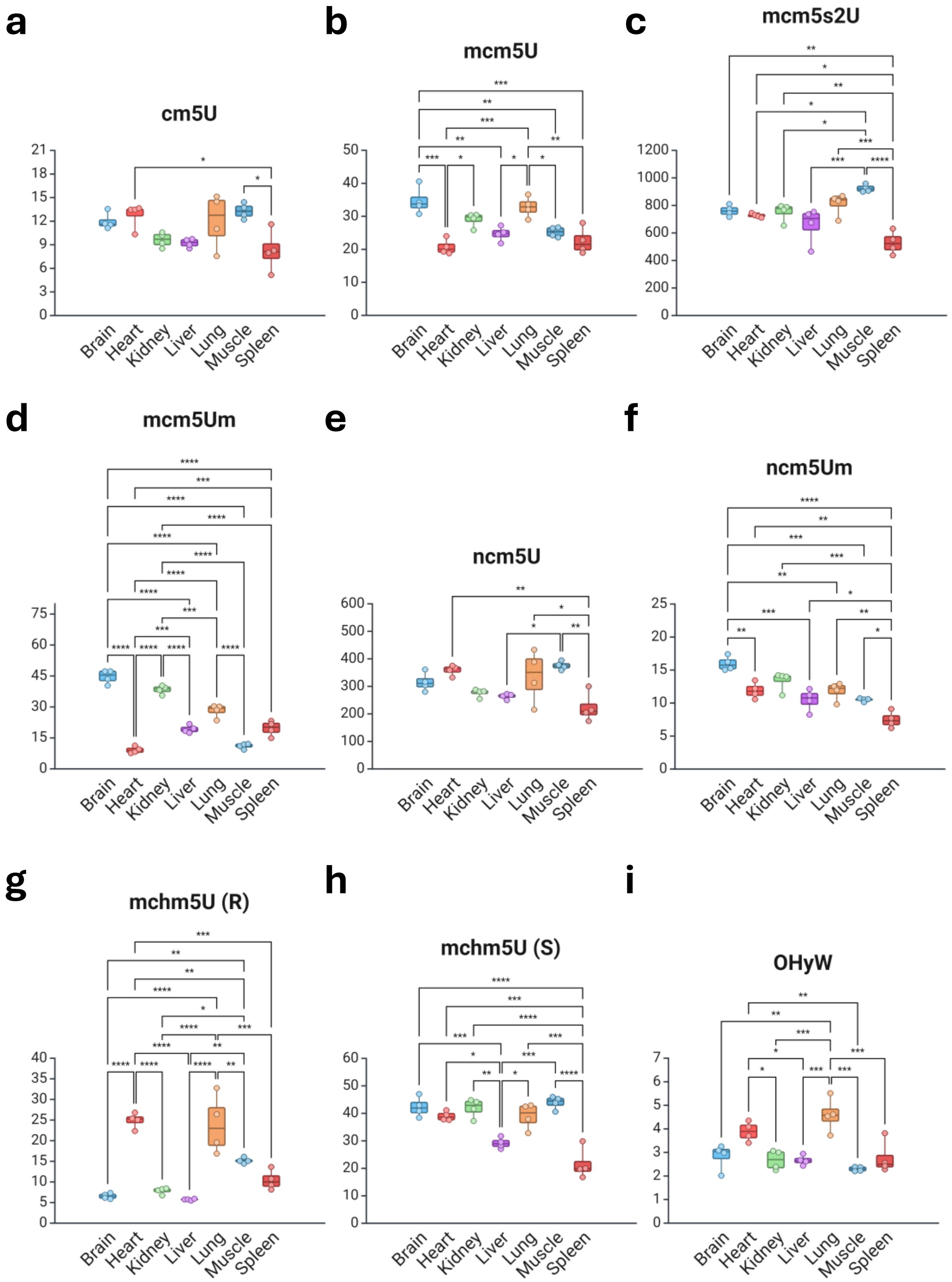
a
cm5U
b
mcm5U
c
mcm5s2U
d
mcm5Um
e
ncm5U
f
ncm5Um
g
mchm5U (R)
h
mchm5U (S)
i
OHyW
Brain
Heart
Kidney
Liver
Lung
Muscle
Spleen

Supplementary figure S6: Detailed analysis of modifications not reported to occur in tRNA. Y-axis represents normalized peak areas. One-way ANOVA was conducted with Turkey's post-hoc analysis. *: $p < 0.05$, **: $p < 0.005$, ***: $p < 0.0005$, ****: $p < 0.0001$.

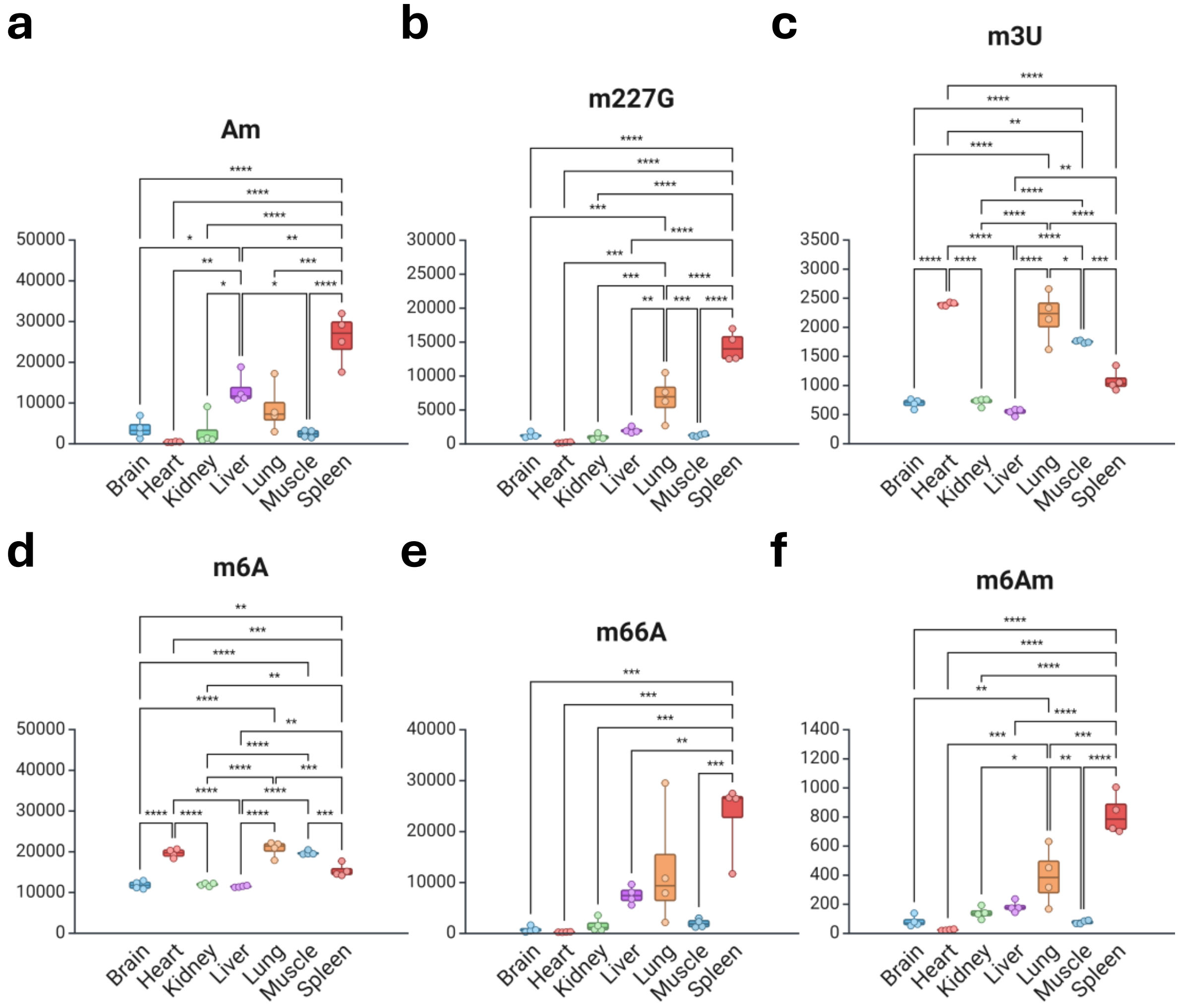

a
Am
b
m227G
c
m3U
d
m6A
e
m66A
f
m6Am
Brain
Heart
Kidney
Liver
Lung
Muscle
Spleen

Supplementary figure S7: Detailed analysis of modifications that occur in tRNA and other non-coding small RNAs. Y-axis represents normalized peak areas. One-way ANOVA was conducted with Turkey's post-hoc analysis. *: $p < 0.05$, **: $p < 0.005$, ***: $p < 0.0005$, ****: $p < 0.0001$.

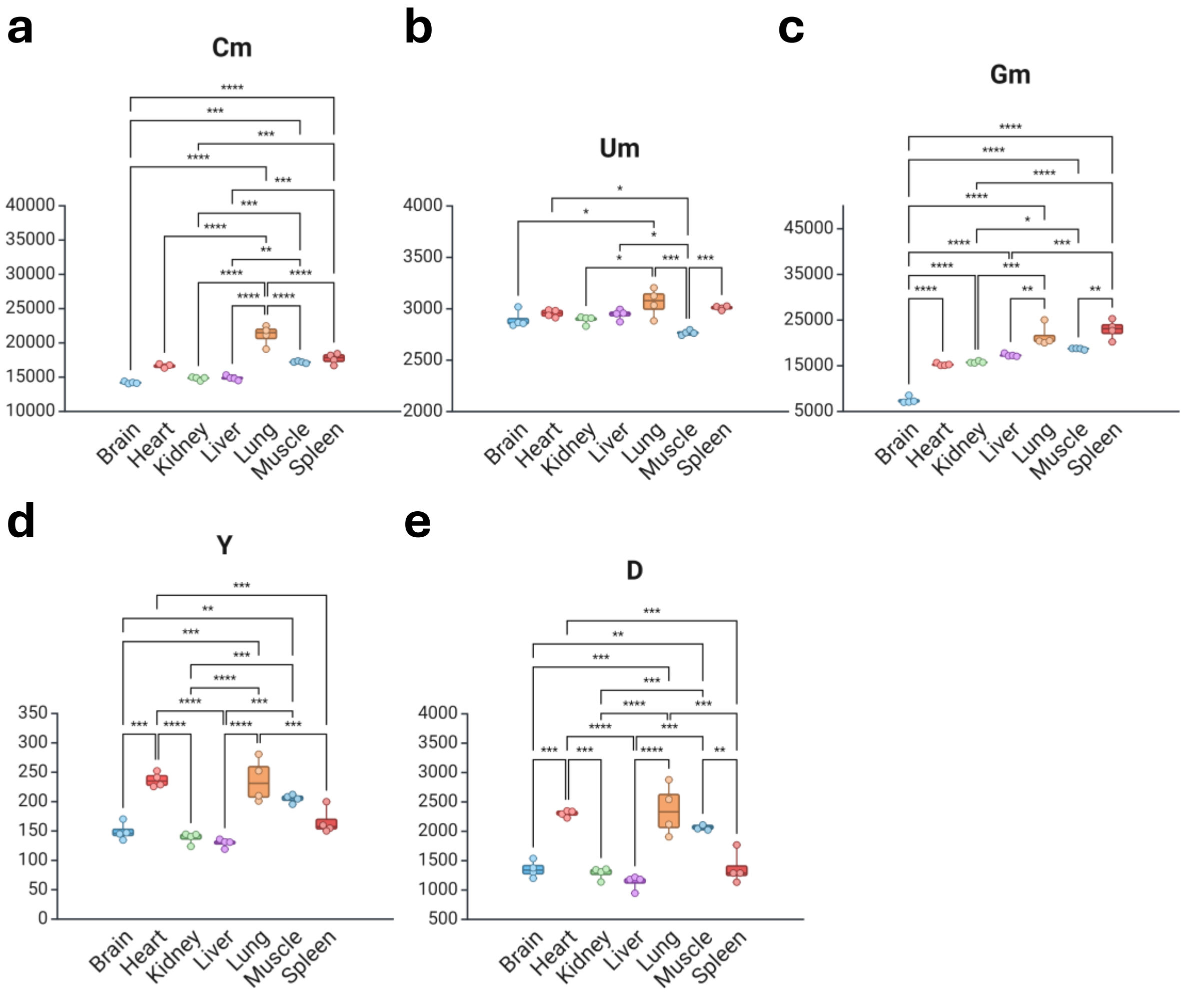

a
Cm
b
Um
c
Gm
d
Y
e
D
Brain
Heart
Kidney
Liver
Lung
Muscle
Spleen

Supplementary figure S8: Detailed analysis of modifications that occur in tRNA and other non-coding small RNAs. Y-axis represents normalized peak areas. One-way ANOVA was conducted with Turkey's post-hoc analysis. *: $p < 0.05$, **: $p < 0.005$, ***: $p < 0.0005$, ****: $p < 0.0001$.

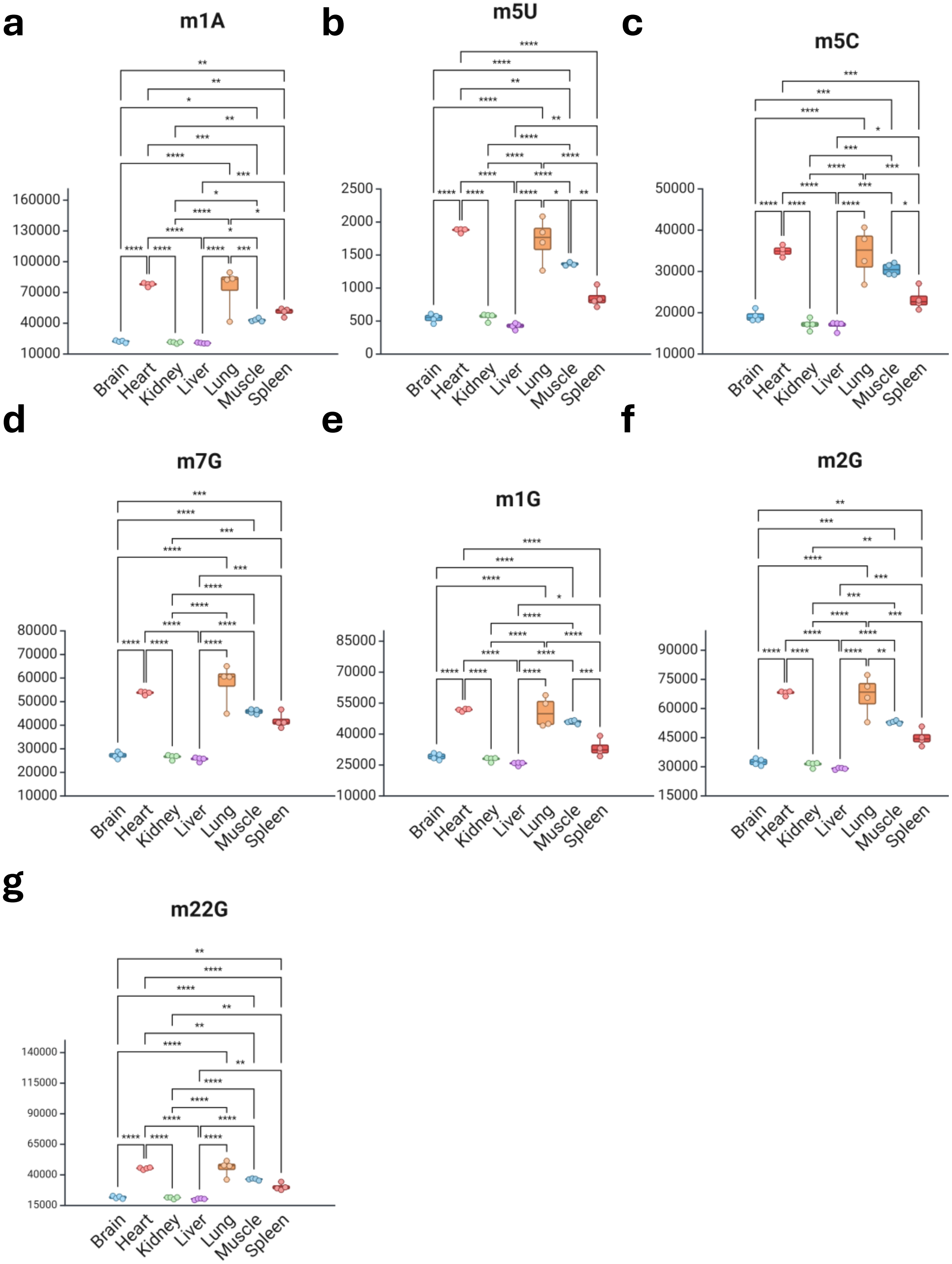
a
m1A
160000
130000
100000
70000
40000
10000
Brain
Heart
Kidney
Liver
Lung
Muscle
Spleen
b
m5U
2500
2000
1500
1000
500
0
c
m5C
50000
40000
30000
20000
10000
d
m7G
80000
70000
60000
50000
40000
30000
20000
10000
e
m1G
85000
70000
55000
40000
25000
10000
f
m2G
90000
75000
60000
45000
30000
15000
g
m22G
140000
115000
90000
65000
40000
15000

Supplementary figure S9: Example bioanalyzer traces showing the higher levels of non-coding RNAs in the spleen compared to tRNA levels.

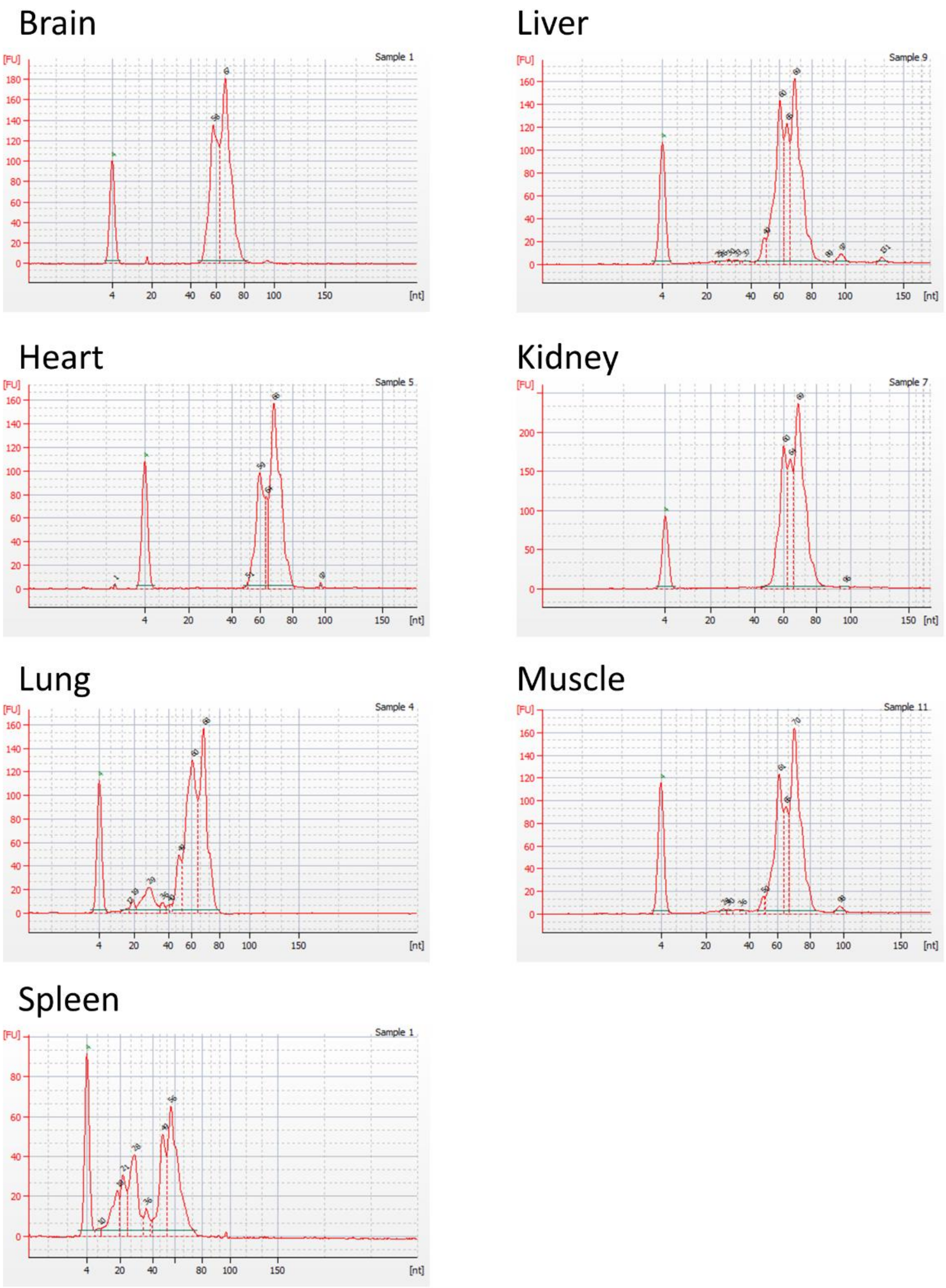

Supplementary figure S10: a: Annotations of heatmap clusters from figure 5a. b: GOBP UMAP clustering. c: Annotations of GOBP UMAP clustering.

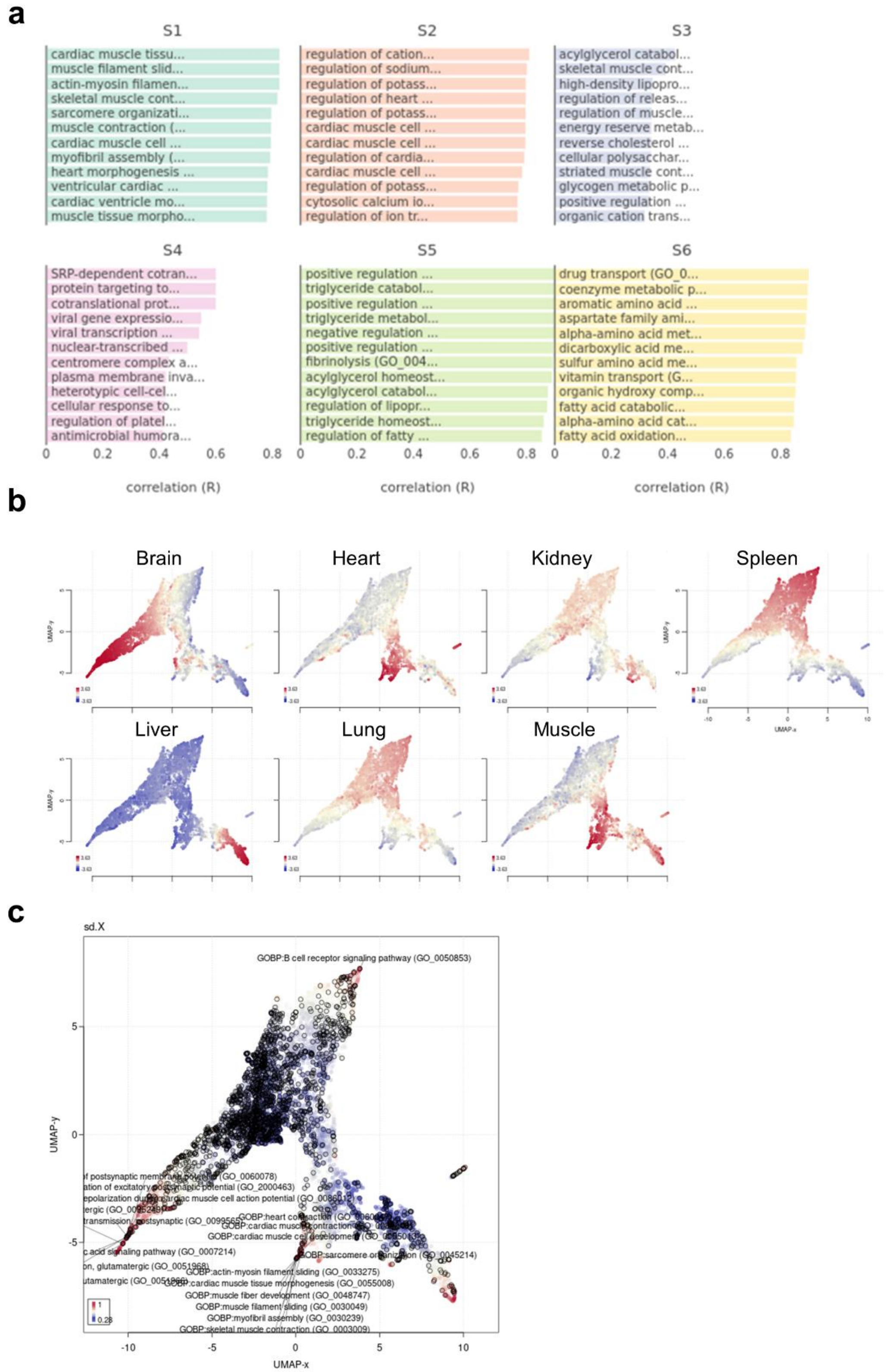

Supplementary figure S11: GOBP activation matrix showing enrichment of pathways when each tissue was compared to all others.

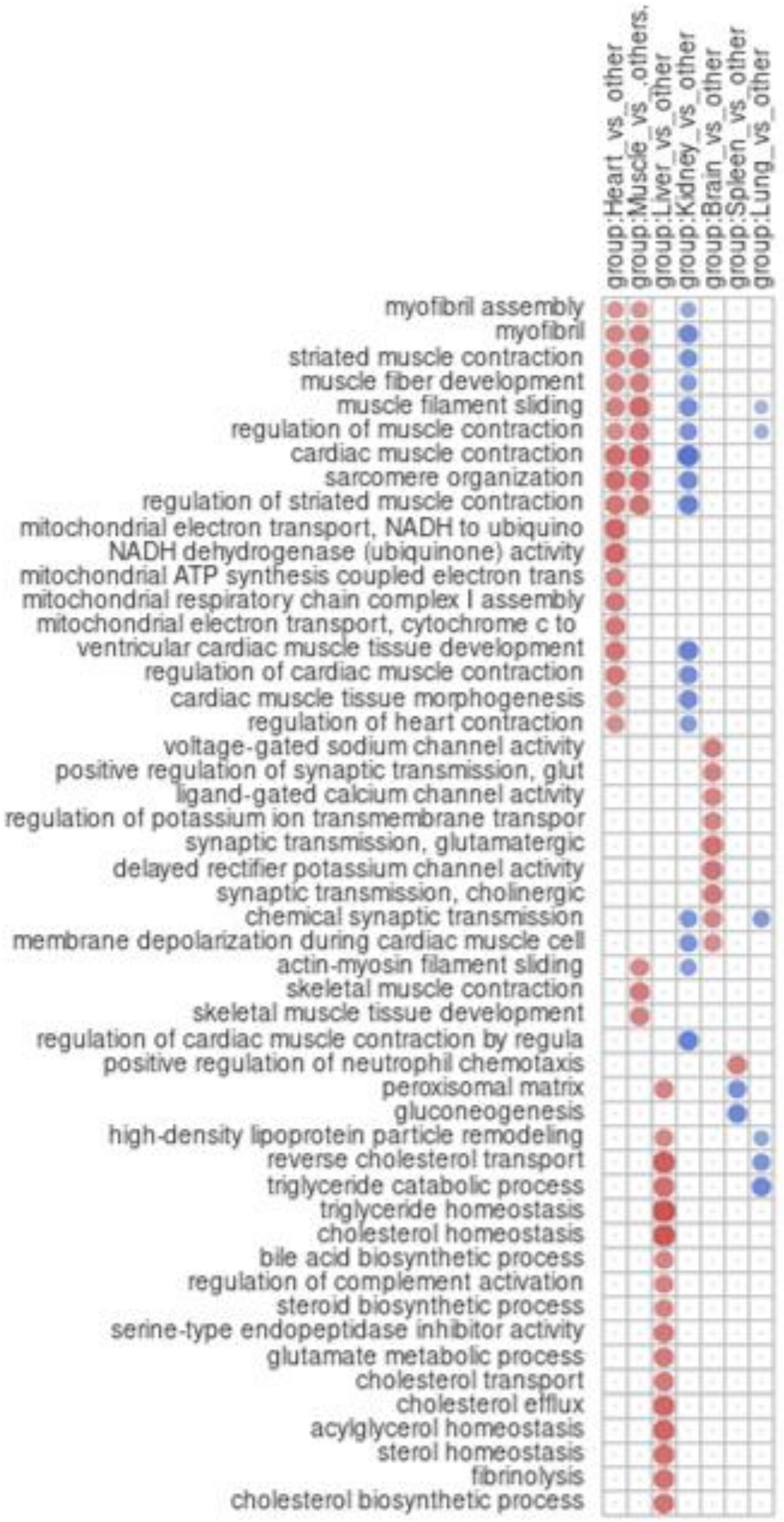

Supplementary figure S12: a: Phenotype (i.e., tissue type) based tSNE clustering. b: Phenotype annotation per sample. c: Phenotype annotation clustering patterns.

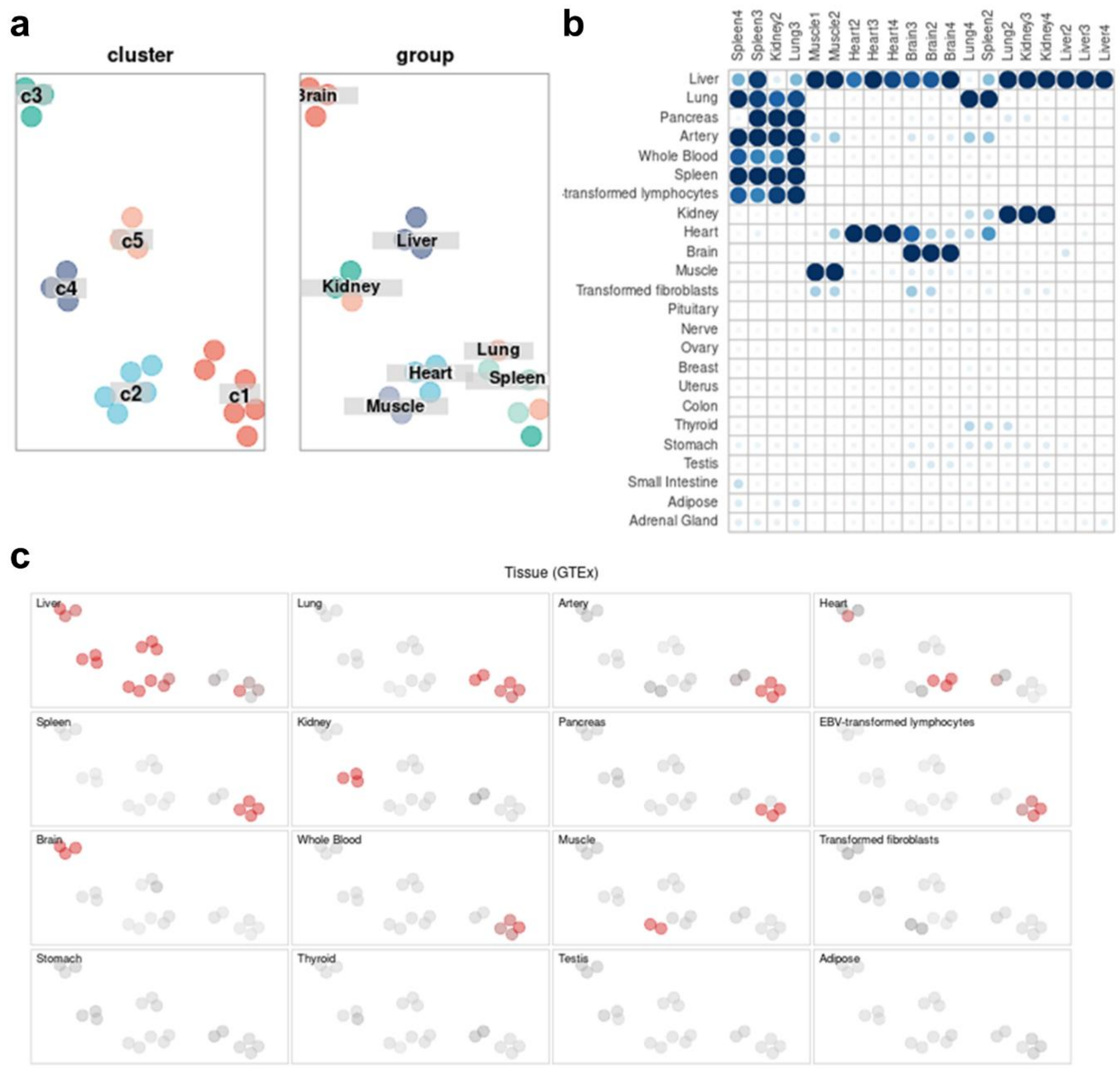

Supplementary figure S13: a: Translation pattern of RNA modifying enzymes in each tissue. b: PLS-DA analysis using the expression of RNA modifying enzymes as input.

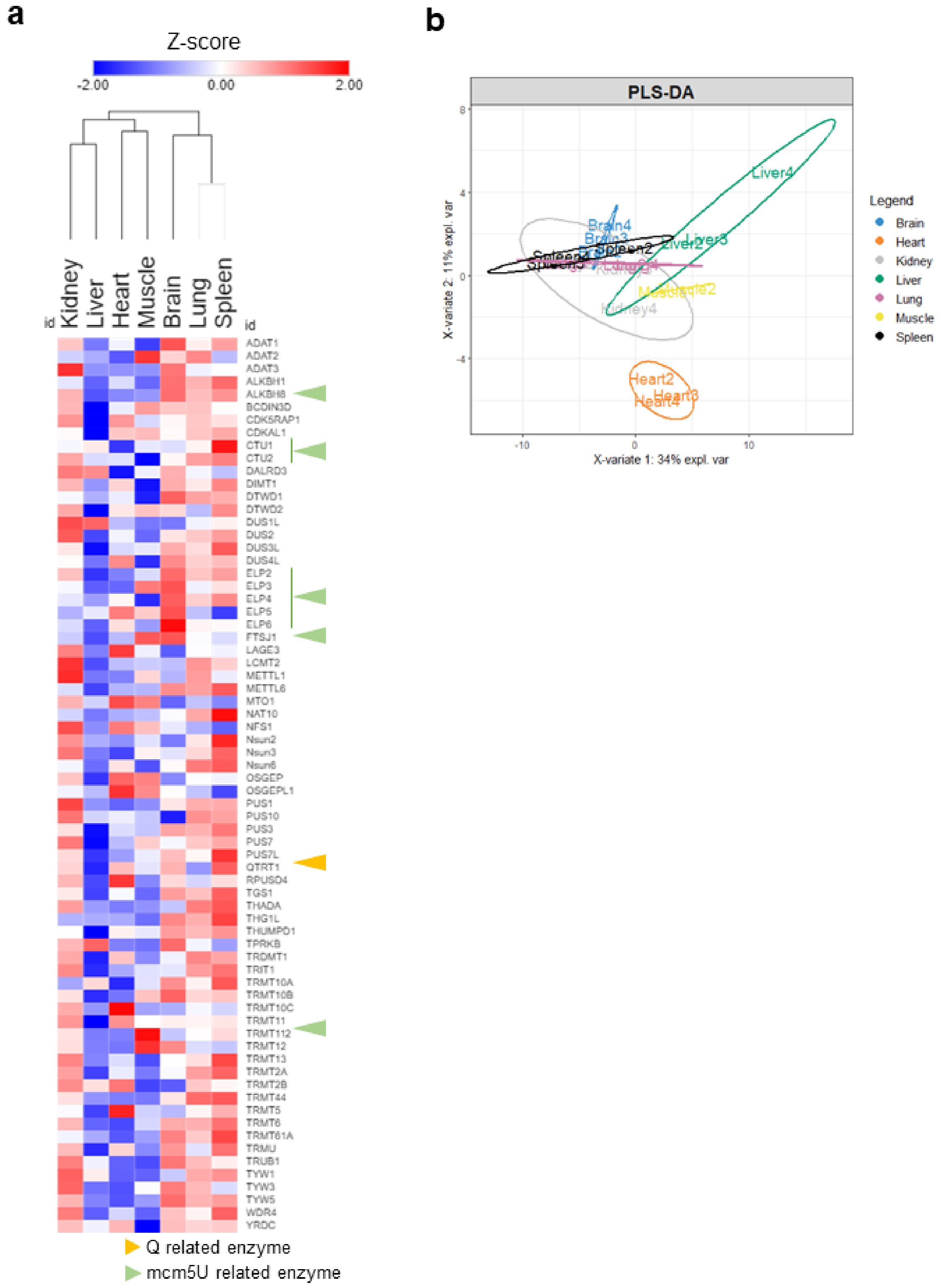

Supplementary figure S14: Different metrics for codon analysis used in this study.

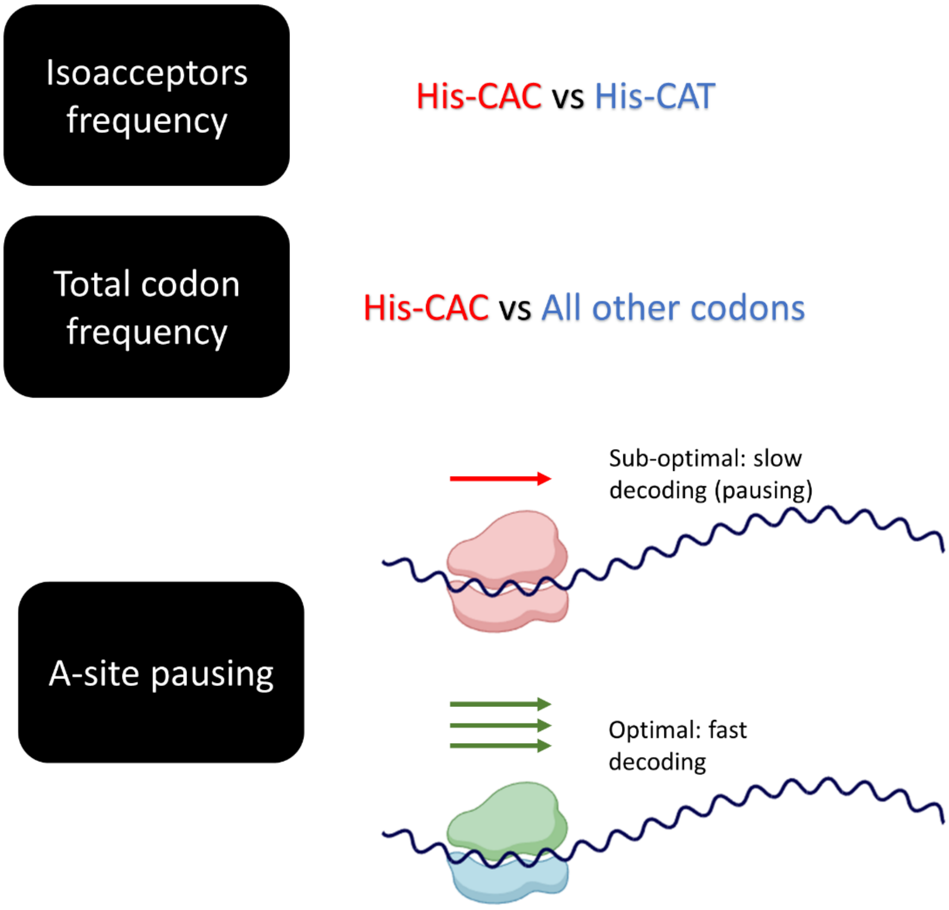

Supplementary figure S15: a: Pairwise correlation analysis (Pearson's correlation coefficient) of all codons from Figure 6a. b: PLS-DA plot of each codon using isoacceptors of the top 200 counted genes.

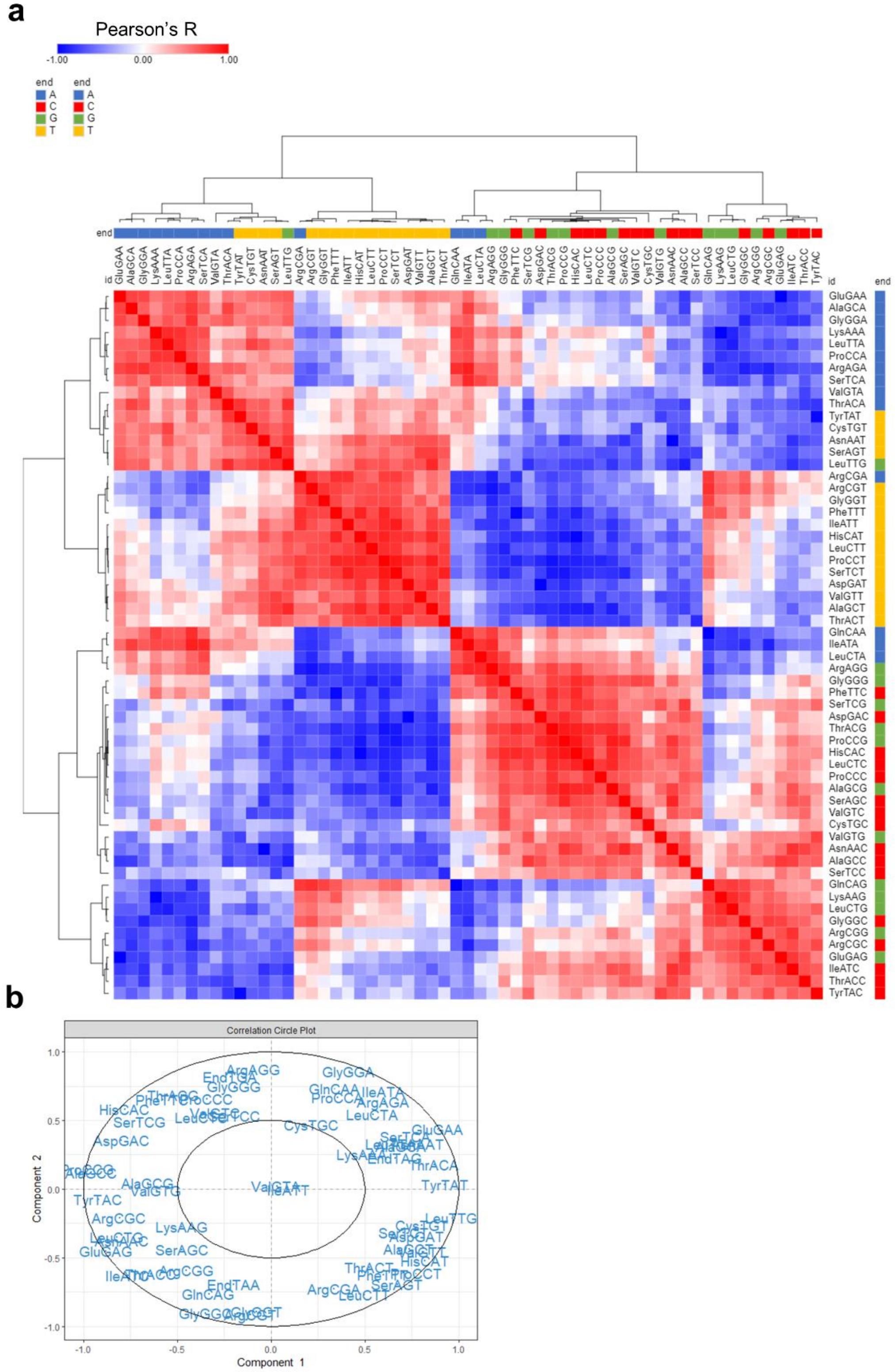

Supplementary figure S16: PLS-DA plot of each codon using total codons of the top 200 counted genes.

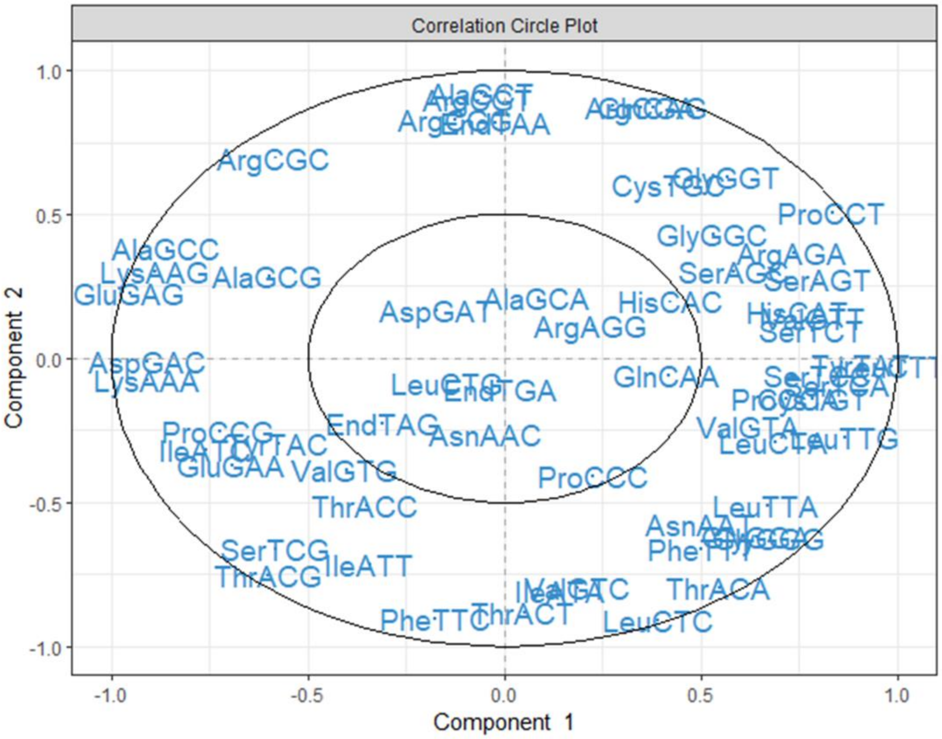

Supplement: Supplementary file 1 [file ijms-26-00706-s001.zip › Supplementary data.pdf]
